# Editorial: Plant diversity and biomass dynamics under environmental variation

**DOI:** 10.3389/fpls.2023.1159695

**Published:** 2023-03-20

**Authors:** Arshad Ali

**Affiliations:** Forest Ecology Research Group, College of Life Sciences, Hebei University, Baoding, Hebei, China

**Keywords:** aboveground biomass, biodiversity, belowground biomass, ecological mechanisms, environmental factors, forests, grasslands

## Background and aims of the research topic

For assessing the long-term viability of grasslands and forests, it is essential to decipher the effects of environmental drivers (including climate and soil) on plant diversity and biomass dynamics (including above-ground and below-ground parts) and their interconnections in both natural and experimental conditions (van der Plas, 2019; Wang et al., 2019; Ma et al., 2021; Cabon et al., 2022). Although plant diversity and composition (including functional traits) are the main biotic drivers of plant biomass dynamics, environmental factors could have significant impacts on both plant diversity and biomass (Díaz et al., 2007; Chu et al., 2016; Poorter et al., 2017; Abbasi et al., 2022). Over the last few decades, most research on plant diversity and biomass has been focused on how niche complementarity, mass ratio, and selection effects play a role in understanding how biodiversity and ecosystem functioning can be connected in natural and experimental plant communities (van der Plas, 2019; Abbasi et al., 2022). Yet, exploring the pathways by which plants interact with their environment is important in understanding how they grow and produce energy which in turn can affect plant diversity and biomass (Chu et al., 2016; Poorter et al., 2017; Michaletz et al., 2018).

**Figure 1:**
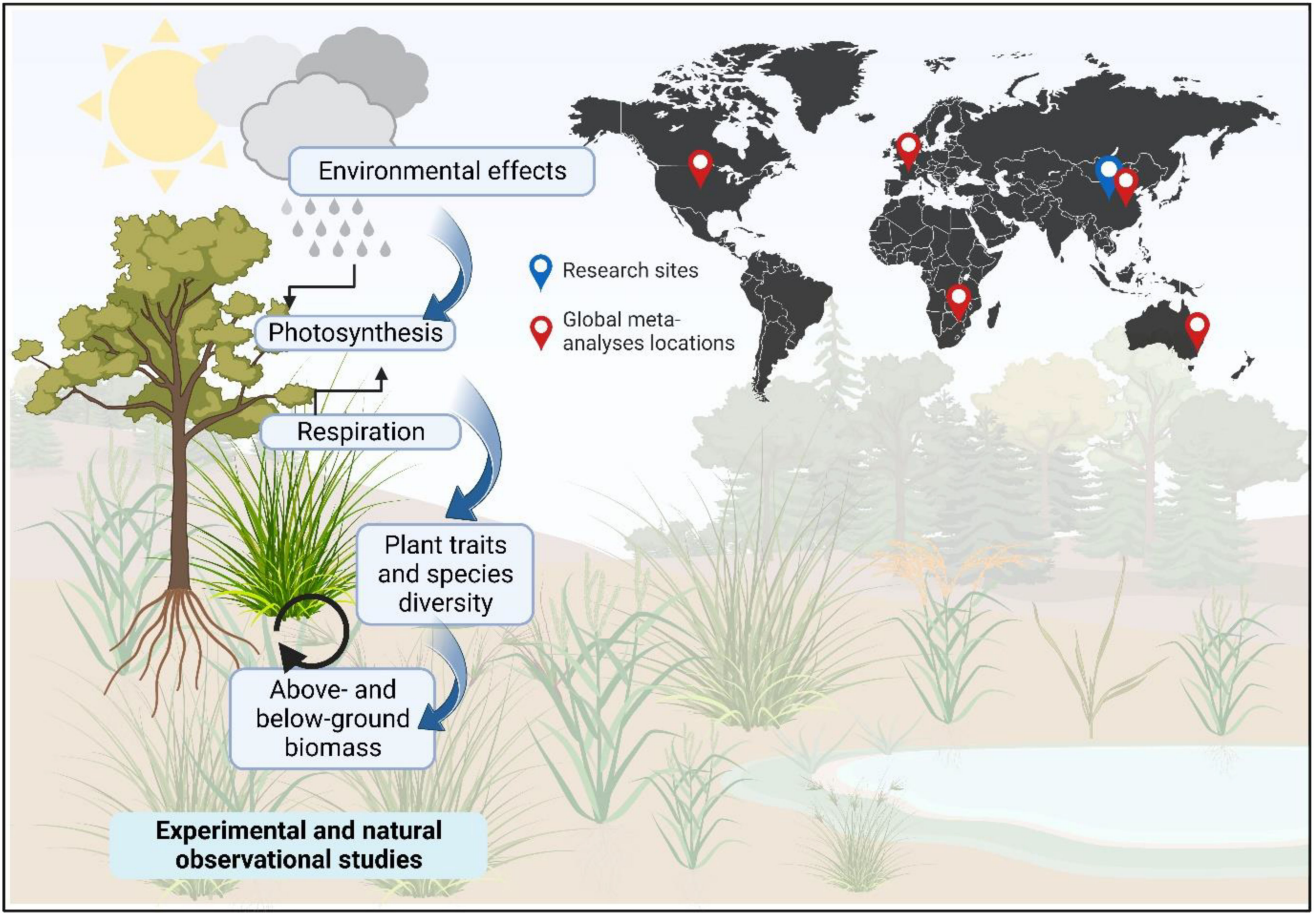
Conceptual framework showing the scientific scope of this Research Topic (e-book).

Understanding the environmental effects on biotic processes is essential for predicting how climate change will affect ecological processes that have feedback on the plant’s physiological processes (**Figure 1**; left side) (Chu et al., 2016; Michaletz et al., 2018; Cabon et al., 2022). The plant physiology and ecology concepts suggest that climate factors, such as temperature and precipitation, have several divergent and convergent influences on the productivity and functioning of ecosystems (Corlett, 2016). The photosynthetic rates, respiration rates, and the distribution of plant biomass are the key physiological processes that determine plant development and are directly influenced by temperature and water availability (Brown et al., 2004; Huxman et al., 2004; Chu et al., 2016; Abbasi et al., 2022). Also, climate can influence plant biomass indirectly *via* adjusting the biodiversity and structure of a community or ecosystem (Poorter et al., 2017; Michaletz et al., 2018; Ma et al., 2021). Many studies have shown that plants suffer more from climate change because of drought and heat which govern the diversity and distribution of plants and the dynamics of their biomass (Corlett, 2016; Bennett et al., 2021; Abbasi et al., 2022). However, it is generally suggested that advancement in research occurs when theoretical and natural observational studies coincide with experimental studies (such as environmental manipulation) (Loreau et al., 2001; Díaz et al., 2007). While researching from both natural observational and experimental viewpoints, we are still not sure if the effects of the environment on plant diversity and biomass are the same for every ecosystem. Furthermore, there is little research on these consequences on a worldwide level.

Our understanding is still limited, thereby demanding additional research across ecosystems and biomes to investigate how environmental factors affect plant diversity and biomass dynamics in natural and experimental plant communities. This Research Topic provided a platform to collect papers on linking the environment, biodiversity and plant above- and below-ground biomass in grasslands and forests through experimental and natural observational approaches. In doing so, I bring together eight theoretical, observational, and experimental studies in China’s grasslands and forests with two global meta-analyses (see general map in **Figure 1**). The ten published papers have tested the research questions using advanced statistical models to demonstrate and discuss the drivers and mechanisms of plant diversity and biomass dynamics under experimental environment control and natural conditions.

## Contributions of the research topic

**Figure 2:**
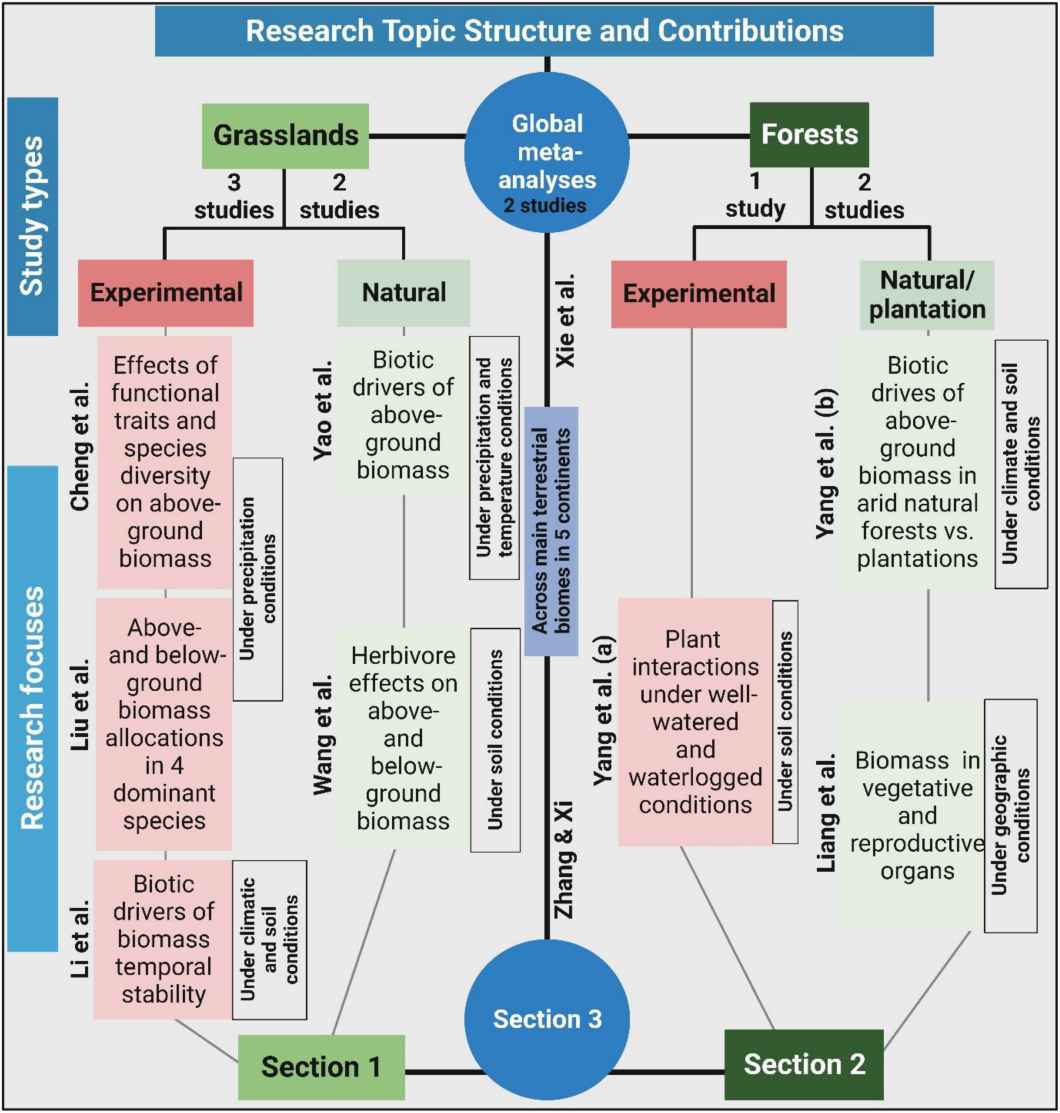
Flowchart diagram showing the structure and contributions of this Research Topic (e-book).

Ten publications make up this Research Topic (e-book), including eight original studies from China and two global meta-analyses (see general map in **Figure 1**), thereby encompassing both forests and grasslands (see **Figure 2**). The authors of eight original studies evaluated the effects of environmental conditions on plant diversity and biomass as well as their interrelationships through statistical modellings, using original data from control experiments in grassland (Cheng et al.; Liu et al.; Li et al.) and young tree seedling in a greenhouse (Yang et al.), and natural observations in grasslands (Yao et al.; Wang et al.) and forests (Yang et al.; Liang et al.). The two global meta-analyses (Zhang and Xi; Xie et al.) have studied the terrestrial ecosystems in five continents to explore the effects of precipitation on above- and below-ground biomass as well as biodiversity. For a better understanding of the research findings, this e-book can be divided into three main sections, i.e., experimental and natural studies on grasslands (Section 1; Cheng et al.; Liu et al.; Li et al.; Yao et al.; Wang et al.), studies on forests (Section 2; Yang et al.; Yang et al.; Liang et al.), and global meta-analyses on terrestrial ecosystems (Section 3; Zhang and Xi; Xie et al.) (**Figure 2**). Although the specific research questions of the ten studies included in this Research Topic have investigated the connections between plant diversity and biomass under environmental variation, the broad contributions can be summarized and discussed in the following main points: 1) biotic drivers (e.g., plant traits, coverage, tree sizes and species diversity) of China’s grasslands and forests under environmental controls; 2) effects of precipitation and warming on plant diversity and biomass; and 3) the effects of precipitation on global ecosystem productivity.

### Biotic drivers of China’s vegetation biomass under environmental controls

Although several studies have examined the biotic drivers of above-ground biomass in natural forest and grassland ecosystems, further research is needed to improve our understanding of these relationships in grasslands subjected to either environmental manipulation or covering large-scale, and in the tree communities of arid regions (van der Plas, 2019). In this Research Topic, Cheng et al. show that the community-weighted mean (CWM) of plant height and leaf dry matter content promoted whereas leaf area declined above-ground biomass directly under the precipitation manipulation experiments in the desert steppe of Inner Mongolia, indicating that plant functional trait composition rather than species diversity is the major biotic driver of ecosystem functioning (Díaz et al., 2007; van der Plas, 2019). Moreover, Liu et al. show that, under the growing season precipitation manipulation experiments in the Inner Mongolia steppe, patterns of plant biomass allocation varied significantly between the four dominant grassland species, pointing to morphological differences between plant species, which may explain how biomass is allocated through physiological processes, rates, and scaling rules (Brown et al., 2004; Ma et al., 2021). Interestingly, Li et al. show that, under 9-year soil warming experimental conditions in the Qinghai–Tibetan Plateau, the temporal biomass stability of sedges, which decreased with warming, explained more than two-thirds of the variance in the temporal stability of the entire plant community, indicating that dominant plants better control ecosystem functions than species richness (Díaz et al., 2007; van der Plas, 2019). These experimental studies in Research Topic (Cheng et al.; Liu et al.; Li et al.) show that, under various environmental conditions in grasslands, plant functional traits and dominant functional groups are better predictors of above-ground biomass and even temporal stability than species richness (Díaz et al., 2007; Isbell et al., 2009).

By using the observation data from 123 grassland meta-sites dominated by *Leymus chinensis*, Yao et al. show that while species richness had negligible effects on above-ground biomass across topographical and climatic gradients, plant coverage promoted and species evenness restricted above-ground biomass (Chu et al., 2016; Grace et al., 2016; Sanaei et al., 2019). However, Wang et al. show that the above- and below-ground biomass of the alpine meadows decreased when plateau pika was present, indicating that herbivore disturbance declines biomass probably due to directly altering plant coverage and species diversity (Archibald et al., 2019; Sanaei et al., 2019). By using the greenhouse experiment on two tree species under well-watered and waterlogging treatments, Yang et al. indicate that biotic interactions shape tree diversity and functioning, thus suggesting that suitable plant species should be selected for plantation and revegetation activities in wetland zones (Paquette and Messier, 2010). In natural forest and planted tree ecosystems in the northwest arid region of China, Yang et al. demonstrate that large plants, species diversity, and tree stand density all increased above-ground biomass in the arid region, which shows that the big-sized trees effect and scaling theory are generalizable to arid regions (Lutz et al., 2018; Ali et al., 2019). These observational studies show that the potential biotic driver of above-ground biomass is plant coverage in grasslands, whereas, in tree ecosystems, large plants do so, indicating that the effects of species richness on ecosystem functioning are overridden by dominant species and functional groups (Díaz et al., 2007; Ali et al., 2019; Sanaei et al., 2019).

### Precipitation regulates China’s vegetation directly and indirectly

Although the primary biotic drivers of plant biomass dynamics are plant diversity and composition (including their functional traits), both plant diversity and biomass depend heavily on environmental factors (van der Plas, 2019). In this Research Topic, using the precipitation manipulation experiments in the desert steppe of Inner Mongolia in Northern China, Cheng et al. show that plant functional traits (plant height, leaf area, and leaf dry matter content) rather than species diversity regulated variation in above-ground biomass through direct and indirect pathways, thereby suggesting that plant functional traits are mechanistically linking the responses of ecosystem functioning to changing patterns in precipitation (van der Plas, 2019). As such, another study by Liu et al. in Inner Mongolia steppe shows that, by using manipulation experiments based on growing season precipitation, increased growing season precipitation led to a rise in the above-ground, below-ground, and total biomass of four main grassland species, thereby also showing that biomass allocation patterns between species are dependent on the amount of precipitation (Brown et al., 2004; Ma et al., 2021). Interestingly, Li et al. show that under 9-year soil warming experimental conditions in the Qinghai–Tibetan Plateau, the relative AGB of grasses and forbs significantly increased and that of sedges decreased irrespective of the soil moisture effects but depended on annual precipitation, indicating that the temporal stability of plant community is largely governed by the few dominant plant functional groups which mediate the responses of ecosystem functioning and stability to environmental conditions (Isbell et al., 2009; Wang et al., 2019). By using the greenhouse experiment on two tree species under well-watered and waterlogging treatments, Yang et al. show that plant competitive interactions that occur in well-watered environments changed to mutualistic interactions in waterlogged environments, indicating that harsh environmental conditions lead to niche facilitation between tree species for high productivity and functioning (Brooker et al., 2008).

By using the observation data from 123 grassland meta-sites dominated by *Leymus chinensis*, Yao et al. show that the above-ground biomass was influenced by precipitation and temperature in several ways, both directly and indirectly *via* the coverage and diversity of plants, indicating that the grasslands of northern China are sensitive to climate change, meaning that an increase in atmospheric heat and a decrease in climatic moisture may decline above-ground biomass (Chu et al., 2016; Grace et al., 2016; Ma et al., 2021). However, Wang et al. show that the above- and below-ground biomass of alpine meadows in the eastern Tibetan Plateau’s responded differently to soil variables at sites with and without plateau pika disturbances, suggesting that grazing and herbivore disturbances in grasslands and meadows should be well-controlled and managed to preserve biodiversity over time (Grace et al., 2016; Archibald et al., 2019; Sanaei et al., 2019). In tree ecosystems of China’s northwest arid region, Yang et al. show that big-sized plants respond differently to climatic humidity and soil fertility, and even to species diversity, thereby highlighting the distinct roles that different environmental factors play in shaping the physiological processes of natural and planted large plants, which control plant diversity and above-ground biomass in arid regions (Lutz et al., 2018; Ali et al., 2019). Moreover, Liang et al. show that dove trees at high latitudes tend to have smaller twigs, and they use more resources for stems and leaves but less for flowers, indicating that dove trees adjust their growth and the distribution of twig biomass in response to environmental changes along a latitudinal gradient (Salazar et al., 2019; Ma et al., 2021).

### Precipitation regulates global ecosystem productivity

The above- and below-ground biodiversity and ecosystem productivity are regulated by environmental factors such as precipitation and temperature, which provide feedback to climate change (Trenberth et al., 2014). However, it has been increasingly recognized that precipitation patterns can have a big impact on ecosystem biodiversity, structure, and functions as it affects climatic and edaphic water availability (Chu et al., 2016; van der Plas, 2019; Abbasi et al., 2022). However, several issues, including (1) assessing patterns of combined responses of soils and plants to climatic conditions, and (2) intra-annual rainfall patterns and their influence on the biodiversity and productivity of terrestrial ecosystems, remain unresolved at a wide geographical scale. In this Research Topic, by using meta-data from 32 global sites, located in Asia, North America, Africa and Oceania, Zhang and Xi show that both above- and below-ground plant biomass as well as soil microbial biomass can react asynchronously to changes in precipitation, thus it is crucial to investigate the plant-soil feedback to comprehend how environmental changes affect grassland ecosystems. In addition, by using meta-data from 19 global sites across 6 major biomes, located in Asia, North America and Oceania, Xie et al. show that the above-ground plant production was enhanced by the regularity of precipitation, while below-ground plant growth was promoted by the varying patterns of precipitation, thus suggesting the importance of precipitation event timing uniformity and heterogeneity on ecosystem functioning.

The two global or large-scale meta-analyses (Zhang and Xi; Xie et al.) included in this Research Topic, contribute several important findings and cover the knowledge gaps for linking above- and below-ground biomass with environmental factors across terrestrial ecosystems. Both meta-analyses agree with the key finding that precipitation is an important environmental factor to regulate the responses of biodiversity and ecosystem productivity to drought and heat, i.e., climate change. Also, Zhang and Xi provide evidence that the asynchrony between above-ground and below-ground production, as well as microbial biomass carbon, is mediated by plant biomass allocation, thereby supporting the optimal allocation theory and emphasising that understanding the consequences of precipitation changes on grassland ecosystems may require understanding the plant-soil feedback (Brown et al., 2004; van der Plas, 2019; Ma et al., 2021). Also, Xie et al. emphasize that not only ecosystem productivity but also community structure and biodiversity respond to changes in precipitation patterns, thereby indicating that the relationship between biodiversity and ecosystem functioning is context-dependent which should be further studied at large spatial scales under different environmental conditions (Poorter et al., 2017; Ali et al., 2019; van der Plas, 2019; Abbasi et al., 2022). The findings of these meta-analyses also agree with both experimental and observational studies, included in this Research topic, conducted in China’s grasslands and forests, thereby supporting the general notion that climatic and soil humidity matter for higher biodiversity and productivity in most cases.

## Concluding remarks and future directions

Most of the ten published studies (particularly the original eight studies), in this Research Topic, focused on the effects of species diversity, however, functional trait identity, plant coverage, the existence of a particular functional group, and big-sized plants were often better predictors of ecosystem productivity (i.e., plant biomass). Furthermore, the majority of research suggests that climatic water availability increased but warming restricted plant diversity and ecosystem productivity, and thus ecosystem stability. Conservation should thus not just support plant diversity and biomass in general but also the environmental factors that favor species with optimal functional traits to jointly enhance those ecosystem diversity, structure, functions, and processes that support human well-being.

Studies in this Research Topic explored the effects of environmental factors on plant diversity and the biomass stock of both above- and below-ground portions of an ecosystem. However, future studies should focus on plant diversity- ecosystem multifunctionality which is a less debated topic in the current global ecological literature. Moreover, to better understand the environmental drivers of plant diversity and biomass dynamics, further research is largely needed to clearly consider the plant’s physiological and metabolic processes in both experimental and large-scale observational studies. Moreover, it is very important to explore the unexplored forest and grassland ecosystems of the world to better understand the consequences of biodiversity loss on ecosystem functioning and productivity under global climate change. For future research in well-explored ecosystems, it is crucial to address advanced research questions using an interdisciplinary approach by combining plant biology and climatology. This can have significant implications for science, practice, and policy to better understand how global climate change and biodiversity loss affect both people and nature.

## Author contributions

The author confirms being the sole contributor of this work and has approved it for publication.

## References

[B1] Abbasi U. A. Mattsson E. Nissanka S. P. Ali A. (2022). Biological, structural and functional responses of tropical forests to environmental factors. Biol. Conserv. 276, 109792. doi: 10.1016/j.biocon.2022.109792

[B2] Ali A. Lin S.-L. He J.-K. Kong F.-M. Yu J.-H. Jiang H.-S. (2019). Big-sized trees overrule remaining trees’ attributes and species richness as determinants of aboveground biomass in tropical forests. Global Change Biol. 25 (8), 2810–2824. doi: 10.1111/gcb.14707 31120573

[B3] Archibald S. Hempson G. P. Lehmann C. (2019). A unified framework for plant life-history strategies shaped by fire and herbivory. New Phytol. 224 (4), 1490–1503. doi: 10.1111/nph.15986 31177547

[B4] Bennett A. C. Dargie G. C. Cuni-Sanchez A. Mukendi J. T. Hubau W. Mukinzi J. M.. (2021). Resistance of African tropical forests to an extreme climate anomaly. Proc. Natl. Acad. Sci. 118 (21), e2003169118. doi: 10.1073/pnas.2003169118 34001597 PMC8166131

[B5] Brooker R. W. Maestre F. T. Callaway R. M. Lortie C. L. Cavieres L. A. Kunstler G.. (2008). Facilitation in plant communities: the past, the present, and the future. J. Ecol. 96 (1), 18–34. doi: 10.1111/j.1365-2745.2007.01295.x

[B6] Brown J. H. Gillooly J. F. Allen A. P. Savage V. M. West G. B. (2004). Toward a metabolic theory of ecology. Ecology 85 (7), 1771–1789. doi: 10.1890/03-9000

[B7] Cabon A. Kannenberg S. A. Arain A. Babst F. Baldocchi D. Belmecheri S.. (2022). Cross-biome synthesis of source versus sink limits to tree growth. Science 376 (6594), 758–761. doi: 10.1126/science.abm4875 35549405

[B8] Chu C. Bartlett M. Wang Y. He F. Weiner J. Chave J.. (2016). Does climate directly influence NPP globally? Global Change Biol. 22 (1), 12–24. doi: 10.1111/gcb.13079 26442433

[B9] Corlett R. T. (2016). The impacts of droughts in tropical forests. Trends Plant Sci. 21 (7), 584–593. doi: 10.1016/j.tplants.2016.02.003 26994658

[B10] Díaz S. Lavorel S. de Bello F. Quetier F. Grigulis K. Robson M. (2007). Incorporating plant functional diversity effects in ecosystem service assessments. Proc. Natl. Acad. Sci. United. States America 104 (52), 20684–20689. doi: 10.1073/pnas.0704716104 PMC2410063 18093933

[B11] Grace J. B. Anderson T. M. Seabloom E. W. Borer E. T. Adler P. B. Harpole W. S.. (2016). Integrative modelling reveals mechanisms linking productivity and plant species richness. Nature 529 (7586), 390–393. doi: 10.1038/nature16524 26760203

[B12] Huxman T. E. Smith M. D. Fay P. A. Knapp A. K. Shaw M. R. Loik M. E.. (2004). Convergence across biomes to a common rain-use efficiency. Nature 429, 651. doi: 10.1038/nature02561 15190350

[B13] Isbell F. I. Polley H. W. Wilsey B. J. (2009). Biodiversity, productivity and the temporal stability of productivity: patterns and processes. Ecol. Lett. 12 (5), 443–451. doi: 10.1111/j.1461-0248.2009.01299.x 19379138

[B14] Loreau M. Naeem S. Inchausti P. Bengtsson J. Grime J. Hector A.. (2001). Biodiversity and ecosystem functioning: current knowledge and future challenges. Science 294 (5543), 804–808. doi: 10.1126/science.1064088 11679658

[B15] Lutz J. A. Furniss T. J. Johnson D. J. Davies S. J. Allen D. Alonso A.. (2018). Global importance of large-diameter trees. Global Ecol. Biogeography. 27 (7), 849–864. doi: 10.1111/geb.12747

[B16] Ma H. Mo L. Crowther T. W. Maynard D. S. van den Hoogen J. Stocker B. D.. (2021). The global distribution and environmental drivers of aboveground versus belowground plant biomass. Nat. Ecol. Evol. 5 (8), 1110–1122. doi: 10.1038/s41559-021-01485-1 34168336

[B17] Michaletz S. T. Kerkhoff A. J. Enquist B. J. (2018). Drivers of terrestrial plant production across broad geographical gradients. Global Ecol. Biogeography. 27 (2), 166–174. doi: 10.1111/geb.12685

[B18] Paquette A. Messier C. (2010). The role of plantations in managing the world’s forests in the anthropocene. Front. Ecol. Environ. 8 (1), 27–34. doi: 10.1890/080116

[B19] Poorter L. van der Sande M. T. Arets E. J. M. M. Ascarrunz N. Enquist B. Finegan B.. (2017). Biodiversity and climate determine the functioning of Neotropical forests. Global Ecol. Biogeography. 26 (12), 1423–1434. doi: 10.1111/geb.12668

[B20] Salazar P. C. Navarro-Cerrillo R. M. Cruz G. Grados N. Villar R. (2019). Variability in growth and biomass allocation and the phenotypic plasticity of seven prosopis pallida populations in response to water availability. Trees-Structure. Funct. 33 (5), 1409–1422. doi: 10.1007/s00468-019-01868-9

[B21] Sanaei A. Li M. Ali A. (2019). Topography, grazing, and soil textures control over rangelands’ vegetation quantity and quality. Sci. Total. Environ. 697, 134153. doi: 10.1016/j.scitotenv.2019.134153 31479909

[B22] Trenberth K. E. Dai A. van der Schrier G. Jones P. D. Barichivich J. Briffa K. R.. (2014). Global warming and changes in drought. Nat. Climate Change 4 (1), 17–22. doi: 10.1038/nclimate2067

[B23] van der Plas F. (2019). Biodiversity and ecosystem functioning in naturally assembled communities. Biol. Rev. 94 (4), 1220–1245. doi: 10.1111/brv.12499 30724447

[B24] Wang Y. Cadotte M. W. Chen Y. Fraser L. H. Zhang Y. Huang F.. (2019). Global evidence of positive biodiversity effects on spatial ecosystem stability in natural grasslands. Nat. Commun. 10 (1), 3207. doi: 10.1038/s41467-019-11191-z 31324792 PMC6642091

